# Both host and parasite non-coding RNAs co-ordinate the regulation of macrophage gene expression to reduce pro-inflammatory immune responses and promote tissue repair pathways during infection with *fasciola hepatica*

**DOI:** 10.1080/15476286.2024.2408706

**Published:** 2024-09-30

**Authors:** Dayna Sais, Sumaiya Chowdhury, John. P. Dalton, Nham Tran, Sheila Donnelly

**Affiliations:** a School of Biomedical Engineering, Faculty of Engineering and Information Technology, The University of Technology Sydney, Ultimo, NSW, Australia; b School of Life Sciences, Faculty of Science, The University of Technology Sydney, Ultimo, NSW, Australia; c Molecular Parasitology Laboratory, School of Natural Sciences, University of Galway, Galway, Ireland

**Keywords:** *Fasciola hepatica*, helminth, non-coding miRNAs, miRNA, lncRNA, macrophages, pro-inflammatory response, immune-regulation, host-parasite interactions

## Abstract

Parasitic worms (helminths) establish chronic infection within mammalian hosts by strategically regulating their host’s immune responses. Deciphering the mechanisms by which host non-coding RNAs (ncRNA) co-ordinate the activation and regulation of immune cells is essential to understanding host immunity and immune-related pathology. It is also important to comprehend how pathogens secrete specific ncRNAs to manipulate gene expression of host immune cells and influence their response to infection. To investigate the contribution of both host and helminth derived ncRNAs to the activation and/or regulation of innate immune responses during a parasite infection, we examined ncRNA expression in the peritoneal macrophages from mice infected with *Fasciola hepatica*. We discovered the presence of several parasitic-derived miRNAs within host macrophages at 6 hrs and 18 hrs post infection. Target prediction analysis showed that these Fasciola miRNAs regulate host genes associated with the activation of host pro-inflammatory macrophages. Concomitantly, there was a distinct shift in host ncRNA expression, which was significant at 5 days post-infection. Prediction analysis suggested that these host ncRNAs target a different cohort of host genes compared to the parasite miRNAs, although the functional outcome was predicted to be similar i.e. reduced pro-inflammatory response and the promotion of a reparative/tolerant phenotype. Taken together, these observations uncover the interplay between host and parasitic ncRNAs and reveal a complementary regulation of the immune response that allows the parasite to evade immune detection and promote tissue repair for the host. These findings will provide a new understanding of the molecular interaction between parasites and host.

## Introduction

Infection with parasitic worms (helminths) is a significant global health issue, with more than 1.5 billion people infected with one or more parasite [1]. While the mammalian immune system is capable of parasite expulsion, during natural infections, host protective immune responses are modulated such that the invading helminths are tolerated, and the tissue damage caused by their migration is managed/repaired [2]. Consequently, sterilizing immunity, a characteristic of infection with most other pathogens (viral and bacteria), is not activated. Accordingly, a consistent feature of mammalian helminth infection is that complete expulsion or killing of all parasites is rarely achieved, there is little evidence of protection against re-infection, and for most, there is no effective vaccine [3–5]. While drug treatments are available, these are limited in number, and due to rapid reinfection within endemic populations, the current strategy of mass drug administration is ineffective in the long term. In addition, the continuous widespread use of these drugs in humans and animals has led to the emergence of resistance [6]. A better understanding of the mechanisms that underpin the regulation of protective immunity will inform the design of much needed anti-helminth treatments/vaccines.

*Fasciola hepatica* infection is an ideal model for characterizing the modulation of host immune responses. The parasite progresses through distinct life stages, each associated with defined host tissue sites, thus allowing analysis of specific interactions between parasite and host immune cells. Infection with this parasite only occurs when the mammalian host ingests water or vegetation contaminated with the infective metacercariae [7]. Within hours after ingestion, the metacercariae excyst to release the infectious newly excysted juveniles (NEJs). The NEJs perforate the intestinal wall to migrate through the peritoneal cavity to reach the liver, where they grow and mature. This activity induces an influx of immune cells, predominantly macrophages, into the peritoneal cavity [8,9]. However, despite the presence of migrating NEJs, the damage to the intestinal epithelium, and the presence of translocating intestinal microbiota, there is a distinct absence of an expected protective pro-inflammatory M1-like macrophage phenotype. Instead, macrophages in the peritoneal cavity of infected animals (mice and sheep) display no significant increase in the production of pro-inflammatory cytokines (TNF, IL-12, IFNγ) or anti-microbial effectors [iNOS, nitric oxide (NO)] [8–11]. As the infection progresses and the NEJs penetrate the liver capsule, M2-like macrophages dominate, as characterized by an increase in the expression levels of Arg1 and Ym1 [12]. The absence of M2-like macrophages during this phase of infection had no effect on the size or number of infecting parasites but did result in more extensive liver damage and premature host death. Therefore, it was concluded that the primary role of the M2-like macrophages during infection with *F. hepatica* (and other helminths) is the promotion of tissue repair rather than mediating anti-helminth responses [13]. The aim of the current study was to define the molecular mechanisms underlying this interplay between parasite and host macrophages.

Non-coding RNAs (ncRNAs), such as miRNAs and long non-coding RNAs (lncRNAs), play vital roles in gene regulation in a multitude of cellular pathways to maintain cellular homoeostasis. NcRNAs are often expressed in response to stimuli and to abnormal changes to a cell and have been observed to exhibit differential expression in numerous diseases [14], including parasitic infections [15–18]. There are several forms of ncRNAs that are categorized according to their size. The small ncRNA group includes microRNAs, which are short single-stranded RNAs around 22nt in length. MicroRNAs bind to complementary sequences in their target messenger RNA (mRNA) 3`untranslated regions (3`UTRs) to induce mRNA degradation and translational repression [19]. Non-coding RNAs that are 200nt or greater in length are referred to as long non-coding RNAs (lncRNAs) [14]. These can form secondary structures, enabling them to interact with DNA, RNA, and protein molecules, thus acting in transcriptional and post-transcriptional regulation and as regulators of chromatin organization [20,21].

As ncRNAs function to regulate gene expression, it is not surprising that both miRNAs and lncRNAs have central roles in macrophage activation, polarization, and resolution of inflammation [22,23]. During helminth infection, changes in the expression of host miRNAs and lncRNAs in multiple tissue sites are documented for most parasites [24–26]. In macrophages, these changes have been predicted [24,26], and experimentally [27–29] shown to have immune-modulating outcomes. Amongst these effects is the regulation of Toll-like receptor signalling, which may be critical to the control of host protective immune responses, as these receptors evolved to act as innate sensors of pathogen ligands [30]. Activation of macrophage TLRs shapes the hosts immune response to infection and plays a pivotal role in regulating the balance between activation of pro-(M1) and anti-(M2) inflammatory macrophages to mediate the survival of both host and parasite [30,31]. During infection, helminth-derived miRNAs are transferred to host macrophages, likely via the uptake of extracellular vesicles (EVs) [29,32–35]. The parasite miRNAs insert into the host miRNA biogenesis machinery, adopting the mammalian mechanism of gene regulation, to alter the inflammatory response of macrophages. While these studies provide evidence that ncRNAs (from host and parasite) [29,33] operate to regulate the biological function of macrophages, most have focused on a single miRNA at a single timepoint or only on the function of host or parasite-derived ncRNAs independently of each other. To date, no study has investigated the combined effect of both host and parasite ncRNAs on macrophage functionality over the course of helminth infection.

Thus, here, we have characterized the complete profile of host and parasite derived ncRNAs in murine peritoneal macrophages during the first five days of infection with *F. hepatica*. These ncRNAs were then matched to their corresponding gene targets within the transcriptome of the same macrophages, which allowed the assembly of ncRNA:mRNA interactome. The analysis revealed the joint influence of both host and parasite in regulating macrophage inflammatory responses over the course of infection.

## Materials and methods

### Murine infections and collection of peritoneal macrophages

BALB/c female mice aged 6–8 weeks of age (Australian Resource Centre, Perth, Australia) were orally infected with 20 metacercariae of *F. hepatica* (Invetus, Australia). Peritoneal exudate cells were collected by washing the peritoneal cavity of mice with sterile saline at 6 hours (hrs), 18 hrs, and 5 days post-infection (*n* = 25/timepoint) and from a cohort of uninfected mice (*n* = 25). Magnetic beads were used to negatively select peritoneal macrophages as per manufacturer’s instructions (Miltenyi, USA). Peritoneal macrophages from five mice at each timepoint were pooled together for RNA isolation, giving a total of five replicates for each timepoint. Ethical approval for this study was granted by the University of Technology Sydney (UTS) Animal Care and Ethics Committee (ETH20–4709), and experiments were conducted in accordance with the approved guidelines to be compliant with The Australia Code for the Care and Use of Animals for Scientific Purposes.

### RNA sequencing and bioinformatics analysis

Total RNA was isolated from the peritoneal macrophages using RNAzol RT (Molecular Research Centre, US) as per the manufacturer’s instructions and quantified using a Nanodrop ND 1000 (Thermo Fisher Scientific). Library preparation of RNA from macrophages was performed using the QIAseq prep for miRNA seq (75 M reads/sample) and Illumina Stranded total RNA prep for Total RNA seq (35 M reads/sample) for use in sequencing using Illumina NovaSeq 6000 (The Ramaciotti Centre for Genomics, UNSW, Australia). The quality of the miRNA seq and total RNA seq raw Fastq files was assessed using FastQC v0.11.9, and adapters were removed using Cutadapt v1.12 [36].

For miRSeq fastq files, reads with lengths smaller than 18bp and greater than 26bp were discarded also using Cutadapt. The mature miRNA sequences of *Mus musculus* were retrieved from miRbase (Release 22.2) and were used to build the index for alignment using Bowtie2 [37] and miRSeq reads were aligned to the mature *M. musculus* miRNAs. MiRNA seq reads that did not align against the *M. musculus* miRNAs were then aligned against all published miRNA sequences for *F. hepatica* (Supplementary Table S1). Counts for *M. musculus* and *F. hepatica* miRNAs were extracted from the resultant Sam files using SamTools [38].

For Total RNA sequencing the *Mus musculus* genome sequences (GRCm39.genome.ensembl release 108) were retrieved from Gencode (Release M31) and was used to build the index for alignment using Bowtie2. Counts for *Mus musculus* mRNA and lncRNAs were extracted using Gencode (Release M31) annotation files and HT-Seq count [39].

Raw reads from *Mus musculus* miRNA, mRNA and lncRNAs were analysed for differential expression (DE) using the DESeq2 Bioconductor package (https://bioconductor.org/packages/release/bioc/html/DESeq2.html). Accordingly, raw counts were extracted from all peritoneal macrophage samples, and DESeq2 was employed to normalize counts and identify differentially expressed *Mus musculus* mRNA, lncRNA and miRNAs between uninfected mice and each individual time point (6 hr, 18 hr 5 days) post-infection. One replicate (rpt4) of samples isolated at 18hrs displayed counts for all RNA species much greater than other replicates. This was considered an outlier and subsequently removed from differential expression analysis. Only transcripts (mRNA, miRNA, lncRNA) with a sum of ≥ 10 counts across all samples and with an adjusted p-value <0.05 were included in subsequent analyses. For lncRNAs and miRNAs, a Log2 Fold change of 2 (Log2FC2) or 4-fold was considered for further analyses (predicted target and interactome analyses).

All DE mRNAs were also matched and filtered to the InnateDB database (https://www.innatedb.com/) to identify those *M. musculus* genes are associated with the innate immune response and are altered during *F. hepatica* infection at each time-point.

### Non-coding RNA target prediction

Target prediction analysis was conducted for selected *F. hepatica* miRNAs (fhe-miR-125b-5p, fhe-miR-125a-5p, fhe-miR-71a-5p, fhe-miR-277b-3p) and *Mus musculus* miRNAs (Log2FC2 at one timepoint vs control *n* = 37) and lncRNAs (Log2FC2 across all timepoints *n* = 33). To determine possible mammalian gene targets for miRNAs from both parasite and mouse, the online prediction tools miRDB (miRDB.org), MiRanda on miRNAconsTarget (https://arn.ugr.es/srnatoolbox/amirconstarget/) and TargetScan (http://www.targetscan.org) were utilized. Predictive miRNA:mRNA pairings were filtered for a target score of > 60 in miRDB, target score > 150 and minimum free energy (MFE) <-20k/Cal (Energy-kcal/Mol) in miRanda and a seed sequence match to 7mer-m8 site in TargetScan. All mRNA targets from the three databases were merged and filtered for genes also identified to be differentially expressed in the peritoneal macrophage RNA sequencing data. The miRNA:mRNA pairings were then filtered for those with an inverse expression pattern, i.e. for upregulated miRNAs:downregulated mRNA pairs and for downregulated miRNAs:upregulated mRNA pairs.

As lncRNAs can function as miRNA sponges, we predicted lncRNA:miRNA interactions using Diana tools LncBase.v3 (https://diana.e-ce.uth.gr/lncbasev3) and the predictive miRanda parameters on miRNAconsTarget were used. Again, lncRNA:miRNA interactions found using miRanda were filtered according to the target score > 150 and MFE and <-20k/Cal.

Gene targets of the differentially expressed lncRNAs were then filtered for those with an inverse expression relationship to mRNA transcripts in peritoneal macrophages. Predictive analysis of the DE lncRNAs regulation of genes was performed using the online tools LncRRisearch (http://rtools.cbrc.jp/LncRRIsearch/) and CatRAPID omics v2 (http://s.tartaglialab.com/page/catrapid_omics2_group). LncRNA:mRNA pairs were filtered for an energy threshold of −16 kcal/mol in LncRRisearch, and a binding score cut off > 20 for CatRAPID. As lncRNAs can promote and impede mRNA expression levels, lncRNA:mRNA pairs were filtered for mRNAs differentially expressed in the peritoneal macrophages.

### Interactome network analysis

The predicted miRNA (*F. hepatica and M. musculus*) to mRNA, lncRNA:miRNA and lncRNA:mRNA pairs were imported into the network mapping program Cytoscape v3.9 (https://cytoscape.org/) and merged for a comprehensive network analysis of all RNA interactions occurring during *F. hepatica* infection. All RNA interactions were divided into the corresponding timepoint post-infection (6 hr, 18 hr, D5), at which they were differentially expressed and filtered for those genes associated with the innate immune response using InnateDB.

### Gene ontology and KEGG analysis

Gene ontology analysis was performed on all differentially expressed mRNAs at each timepoint, as well as those filtered through InnateDB. Gene ontology was performed using G:profiler (https://biit.cs.ut.ee/gprofiler/gost), and those statistically significant enriched terms were visualized using Revigo (http://revigo.irb.hr/) r-scripts for the scatterplot.

## Results

### Host ncRNA expression in peritoneal macrophages significantly increases when juvenile *Fasciola hepatica* enters the liver

To decipher the impact of *F. hepatica* on the non-coding RNA landscape of host immune cells during the early stages of infection, peritoneal macrophages were harvested from BALB/c mice infected with *F. hepatica* at 6 hrs, 18 hrs and 5 days post-infection. These time points were chosen to represent the early migration of the flukes from the initial translocation from the intestine (6 hrs), through the peritoneal cavity (18 hrs) to penetration of the liver (5 days). RNA harvested from these macrophages was subjected to total RNA and small RNA sequencing. The miRNAseq reads were cleaned and then aligned to the *M. musculus* miRNAs listed on miRbase. Differential expression analysis was performed with DESeq2 and miRNA expression at each timepoint during infection was compared to miRNA expression in macrophages isolated from uninfected mice (Supplementary Table S2.1).

**Figure 1.**
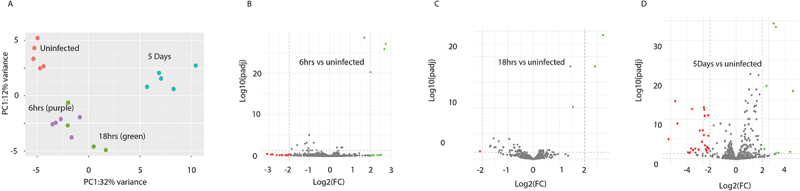
Differentially expressed murine miRNAs in peritoneal macrophages of mice infected with *F. hepatica*. (A) Principal component analysis (PCA) plot of the total murine miRNA expression across all timepoints that were analysed; uninfected (purple), and 6 hr (blue), 18 hr (red) and 5 days (green) post infection. (B-D) volcano plots of differentially expressed murine miRNAs at (B) 6 hr, (C) 18 hr and (D) 5 days post infection as compared to miRNA expression in the macrophages of uninfected mice using DESeq2. Grey points indicate those miRNAs that do not meet the threshold of significance of < 0.05 adjusted *p* value and < ±2log2 fold change (FC). MiRNAs denoted green and red indicate those that are increased or decreased in expression respectively.

**Figure 2.**
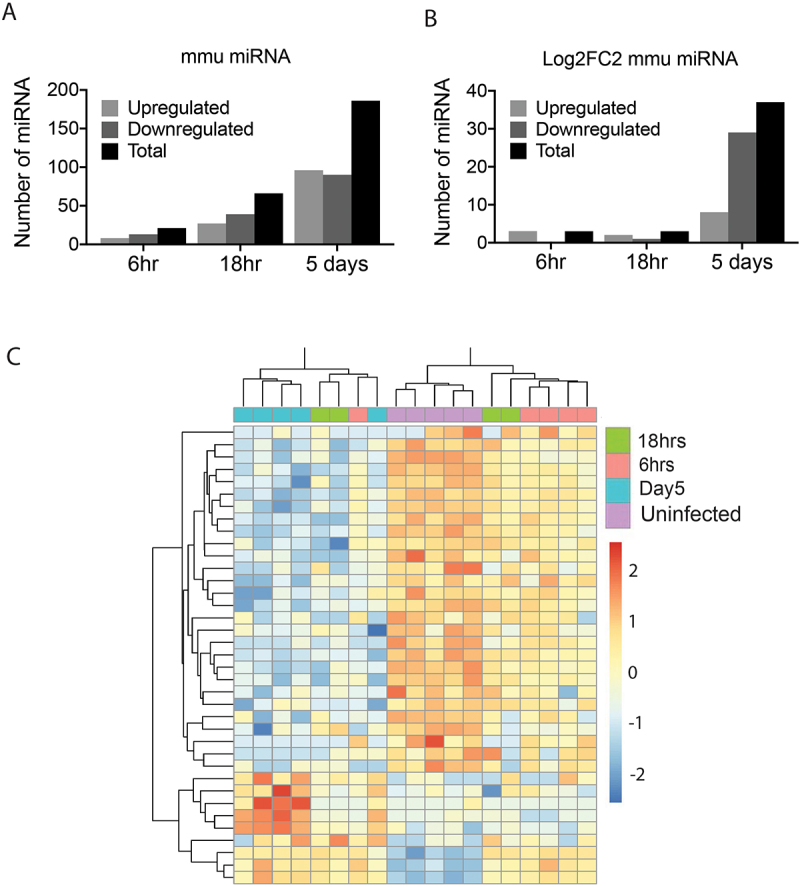
Comparison of murine miRNAs profiles in the peritoneal macrophages of mice show the greatest differential expression at 5 days after infection with *fasciola hepatica*. (A) The total number of differentially expressed (DE) mmu-miRNAs with a < 0.05 adjusted *p* value, in macrophages at each timepoint after infection as compared to uninfected animals. (B) The total number for DE mmu-miRNAs between macrophages from infected mice versus uninfected mice, with a < 0.05 adjusted *p* value and < ±2log2 fold change (4-Fold change). (C) A constructed heatmap displaying the differentially expressed host miRNAs between macrophages isolated from uninfected BALB/c mice (purple) and macrophages from mice infected with *fasciola hepatica* harvested at 6 hours (pink), 18 hours (green) and 5 days (blue) post-infection. The heatmap represents all miRNAs with a fold change greater than or equal to 4-fold for at least one timepoint vs uninfected. MiRNAs shown in red are upregulated, whilst blue are downregulated.

The profile of miRNA expression in macrophages taken from infected mice were distinct from macrophages from uninfected mice. Within the infection time points, the changes in miRNA expression at day 5 were quite different from the profile of differentially expressed (DE) miRNAs observed at the earlier time points of 6 hrs and 18 hrs (Figure 1a). Furthermore, the expression pattern of DE miRNAs in macrophages at both 6 hrs and 18 hrs, were more similar to that seen in the macrophages isolated from uninfected mice (Figure 2). These variations between time points are reflected in the number of miRNAs that were differentially expressed at each time point compared to the miRNA expression in uninfected animals (Figures 1 and 2). In total, 21 DE miRNAs (8 upregulated and 13 downregulated) were detected at 6 hrs, 66 (27 upregulated and 39 downregulated) at 18 hrs, and 189 (96 upregulated and 90 downregulated) at 5 days post-infection (Figure 2a). To identify the most significant mmu-miRNAs during *F. hepatica* infection, a 4-fold cut-off (Log2FC2) was applied, which revealed only 3 DE miRNAs at 6 hrs and 18 hrs, and 37 miRNAs at 5 days post-infection (Figure 2b,c; Supplementary Table S2.2).

Investigating the chromosomal location of the DE miRNAs (with a 4-fold cut-off) revealed that 22 of the 28 downregulated miRNAs altered at 5 days post-infection were clustered on chromosome 12 (Supplementary Table S2.3). MicroRNA clusters are evolutionarily related and are often co-regulated or co-transcribed [40]. Typically, these clusters contain multiple miRNAs, usually transcribed together by a common factor or signalling trigger, resulting in a co-ordinated regulation of functionally related genes.

**Figure 3.**
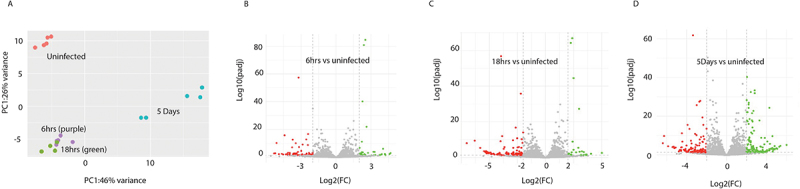
Differentially expressed murine lncRNAs in the peritoneal macrophages of mice infected with *F. hepatica*. (A) Principal component analysis (PCA) plot of the total murine lncRNA expression across all analysed samples; uninfected (purple) 6 hr (blue), 18 hr (red) and 5 days (green) post infection. (B-D) volcano plots of differentially expressed murine lncRNAs at (B) 6 hr, (C) 18 hr and (D) 5 days post infection as compared to lncRNA expression in the macrophages of uninfected mice using DESeq2. Grey points indicate those lncRNAs that do not meet the threshold of significance of < 0.05 adjusted *p* value and < ±2log2 fold change (FC). LncRNAs denoted green and red indicate those that are upregulated and downregulated respectively.

**Figure 4.**
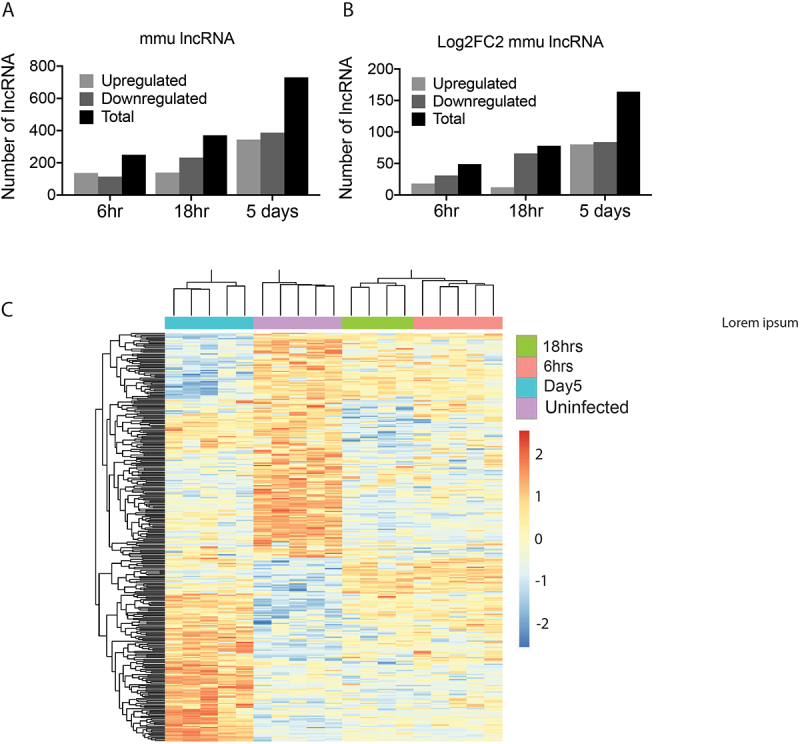
Comparison of murine lncRNAs profiles in the peritoneal macrophages of mice show the greatest differential expression at 5 days after infection with *fasciola hepatica*. (A) The total number of differentially expressed lncRNAs with a < 0.05 adjusted *p* value in macrophages at each timepoint after infection as compared to uninfected animals. (B) The total number lncRNAs in murine macrophages of infected mice that were differentially expressed as compared to uninfected mice by < ±2log2 fold change (4-Fold change) and with a < 0.05 adjusted *p* value. (C) A constructed heatmap displaying the differentially expressed murine lncRNAs between macrophages harvested from uninfected mice (purple) and macrophages at 6 hours (pink), 18 hours (green) and 5 days (blue) post infection with *F. hepatica*. This heatmap represents all lncRNAs with fold change greater than or equal to 4-fold for at least one timepoint vs uninfected. LncRNAs shown in red are upregulated, whilst blue are downregulated.

The total RNA sequencing data was next interrogated to identify the differential expression of *M. musculus* lncRNAs in peritoneal macrophages. Following the same workflow as the miRNA analysis, the total RNA sequencing reads were aligned to the *M. musculus* genome (GRCm39.genome ensembl release 108), and lncRNAs were extracted. Differential expression analysis was conducted using DESeq2, comparing the expression of lncRNAs in macrophages from infected mice to those harvested from uninfected animals (Supplementary Table S3.1). Like the *M. musculus* miRNAs, comparing the profiles of DE lncRNAs showed that the macrophages from uninfected mice presented as a distinct cohort compared to macrophages from all infected mice, and the DE lncRNAs at 6 hrs and 18 hrs clustered together, separate to the DE lncRNAs at 5 days post-infection (Figure 3a). In addition, the pattern of lncRNA expression over the course of the infection also matched the changes in miRNA expression, with an increase in the number of DE lncRNAs over time (Figures 3 and 4) and an early bias of a higher number of downregulated lncRNAs. At 6 hrs post-infection, there were 98 differentially expressed lncRNAs (36 upregulated and 62 downregulated), followed by 116 at 18 hrs (25 upregulated and 91 downregulated) and 227 at 5 days post-infection (120 upregulated and 108 downregulated). Applying a 4-fold cut-off for the DE lncRNAs (Figure 4; Supplementary Table S3.2) resulted in a total of 49 lncRNAs at 6 hrs (18 upregulated and 31 downregulated), 78 at 18 hrs (12 upregulated and 66 downregulated) and 164 at 5 days (80 upregulated and 84 downregulated) post-infection.

### Fasciola-derived miRNAs are detected in host macrophages and are most abundant at 18 hours after infection

Signals from both host and parasite likely contribute to the activation and regulation of macrophages during infection. We have previously demonstrated that *F. hepatica* miRNAs are loaded into the miRNA biogenesis machinery within the macrophages of infected hosts [30]. Therefore, parasite-derived miRNAs present within the peritoneal macrophages during infection were next identified to characterize those that directly regulate the expression of host gene targets and are thus pivotal to influencing host responses. Accordingly, those miRNAs within the total miRNA sequencing data of the peritoneal macrophages that did not align to the mouse miRNAs were then aligned to known *F. hepatica* miRNAs. This analysis identified a total of 29 miRNA sequences *F. hepatica*-specific miRNA sequences (Supplementary Table S4). While no read counts for 28 *F. hepatica* of these miRNA sequences were detected in the macrophages isolated from uninfected mice, the miRNA fhe-miR-novel-11-5p perfectly aligned with the mouse genome and could not be distinguished from the host and was, therefore, excluded from further analysis.

Eight *F. hepatica* miRNAs were detected at 6 hrs, 22 at 18 hrs, and 3 at 5 days post-infection, consistent with our previous findings where *F. hepatica* miRNAs were identified in macrophages at 6 hrs, 12 hrs and 24 hrs but not at day 5 [30]. This expression pattern corresponds with the parasite’s movement through the host; at 6 hrs, the NEJs are migrating through the peritoneal cavity, an activity that would be expected to increase by 18 hrs after infection. During this time, the parasites are most likely interacting with peritoneal macrophages, either directly or via the secretion of EVs containing miRNAs. By day 5, most of the infecting parasites have reached the liver, and therefore, a decrease in parasite-derived miRNAs found within the peritoneal macrophages is expected.

### The transcriptome of peritoneal macrophages from infected mice reveals a significant impact on immune-biological pathways

**Figure 5.**
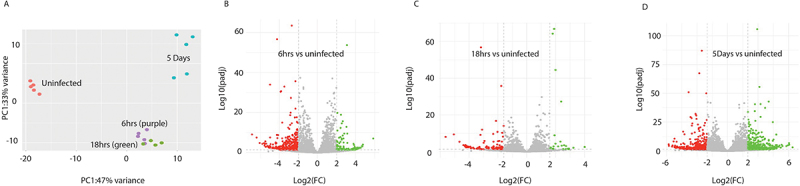
Differentially expressed murine mRNAs in the peritoneal macrophages of mice infected with *F. hepatica*. (A) Principal component analysis (PCA) plot of the total murine mRNA expression across all analysed samples; uninfected (purple) 6 hr (blue), 18 hr (red) and 5 days (green) post infection. (B-D) volcano plots of differentially expressed murine mRNAs at (B) 6 hr, (C) 18 hr and (D) 5 days post infection as compared to mRNA expression in the macrophages of uninfected mice using DESeq2. Grey points indicate those lncRNAs that do not meet the threshold of significance of < 0.05 adjusted *p* value and < ±2log2 fold change (FC). mRNAs denoted green and red indicate those that are upregulated and downregulated respectively.

**Figure 6.**
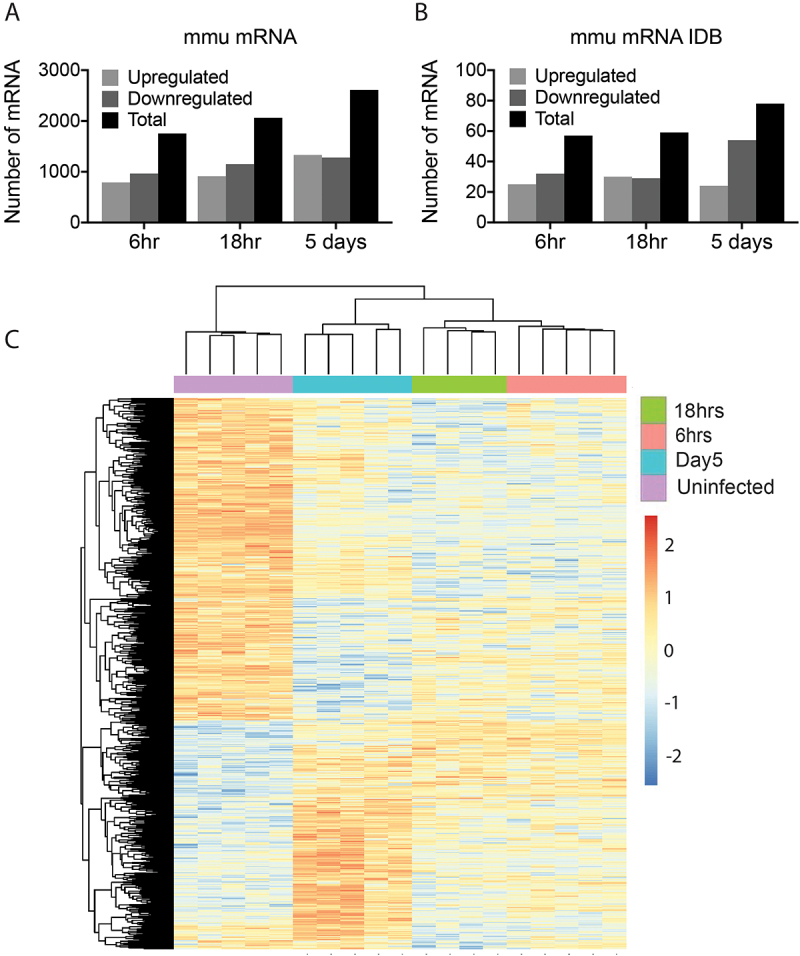
Comparison of murine mRNAs profiles in the peritoneal macrophages of mice show the greatest differential expression at 5 days after infection with *fasciola hepatica*. (A) The total number of differentially expressed mRNAs with a < 0.05 adjusted *p* value in macrophages at each timepoint after infection as compared to uninfected animals. (B) The total number mRNAs in murine macrophages of infected mice that were differentially expressed as compared to uninfected mice by < ±2log2 fold change (4-Fold change) and with a < 0.05 adjusted *p* value. (C) A constructed heatmap displaying the differentially expressed murine mRNAs between macrophages harvested from uninfected mice (purple) and macrophages at 6 hours (pink), 18 hours (green) and 5 days (blue) post infection with *F. hepatica*. This heatmap represents all mRNAs with fold change greater than or equal to 4-fold for at least one timepoint vs uninfected. mRNAs shown in red are upregulated, whilst blue are downregulated.

While the identification of predicted gene targets for both host and parasite-derived ncRNAs can be used to build a hypothesis of a biological outcome, the construction of a ncRNA:mRNA interactome provides a more accurate proposition of functional changes within macrophages. Therefore, the differential expression of mouse (*M. musculus*) mRNAs in peritoneal macrophages at each time-point of infection was compared to cells from uninfected mice. Total RNA sequencing data was aligned to mouse genome sequences (GRCm39.genome ensembl release 108), mRNAs were extracted, and DE analysis was conducted using DESeq2 (Supplementary Table S5.1). The mRNA expression profiles of macrophages from uninfected mice were distinct from the cells harvested from infected animals and matched the distribution of the ncRNAs in peritoneal macrophages. The mRNA profiles at 6 hrs closely resembled those at 18 hrs, with both clustering together (Figure 5a). In contrast, the expression profile of macrophages at day 5 post-infection differed significantly from the earlier time points (Figures 5 and 6). In total, there were 1753 differentially expressed mRNAs at 6 hrs (788 upregulated and 965 downregulated), 2062 at 18 hrs (911 upregulated and 1151 downregulated) and 2611 at 5 days (1330 upregulated and 1281 downregulated).

To determine the impact of the observed transcriptional changes on the cellular pathways that are characteristic of macrophage inflammatory responses, the differentially expressed mRNAs were filtered for their association with innate immunity using InnateDB.com. This identified a comparable number of DE genes at the early time points, with 57 genes at 6hrs (25 upregulated, 32 downregulated) and 59 at 18hrs (30 upregulated and 29 downregulated) post-infection (Supplementary Table S5.2). The number of these DE innate immune genes increased in macrophages at 5 days post-infection to 78, with a disproportionate shift to genes that were downregulated (24 upregulated, 54 downregulated).

**Figure 7.**
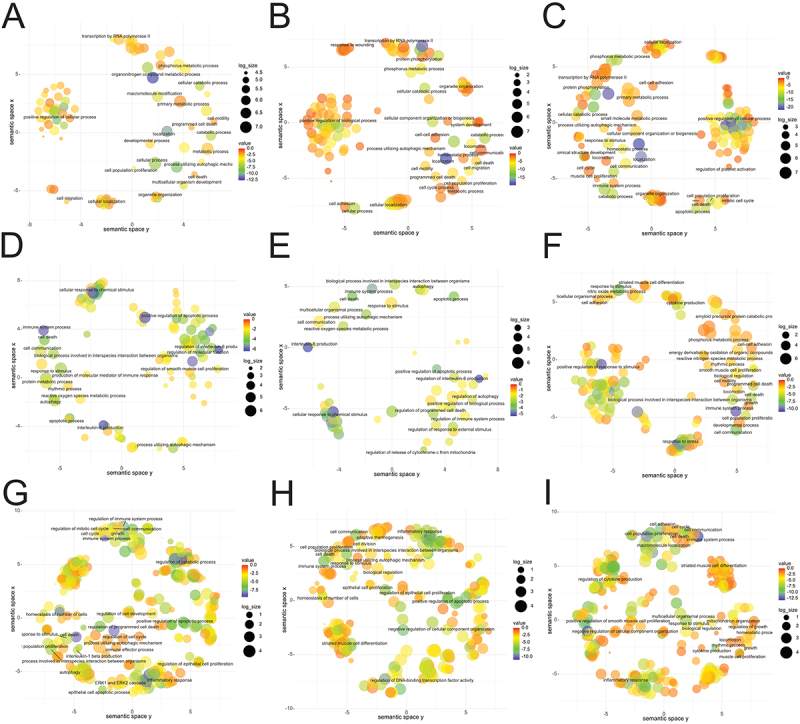
Host and parasite derived ncRNAs regulate the immune pathways of macrophages. Semantic similarity charts on gene ontology enrichment for biological processes associated with differentially expressed genes in the peritoneal macrophages of mice infected with *fasciola hepatica*. (A-C) differentially expressed mRNAs with a < 0.05 p-adjusted value and 2-fold cut-off biological processes at A) 6 hr, B) 18 hr and C) 5 days of infection. (D-F) biological processes of the macrophage genes that are differentially expressed and targeted by host ncRNA (miRNA and lncRNA) at D) 6 hr, E) 18 hr and F) 5 days, filtered for genes listed in IDB. (g-I) biological processes of host macrophage genes that targeted by *fasciola hepatica* miRnas, at G) 6 hr, H) 18 hr and I) 5 days, and filtered for genes listed in IDB. Enrichment analysis was performed using hypergeometric tests and GO terms with < 0.05 adjusted *p* value selected. Charts were constructed using SimRel for represent the semantic similarity measure, where nodes are colour coded based on *p* value and node size represents GO presence.

To characterize the functional impact of this differential expression in mRNAs, a 2-fold change cut-off was first applied to identify the most dysregulated genes, and their cellular functions were then characterized by gene ontology analysis (Figure 7a-c; Supplementary Tables S5.3, S5.4). This analysis revealed that macrophages isolated at 6 hrs after infection had the lowest number of altered biological processes compared to later time points, a not so surprising observation given that these macrophages also displayed the lowest number of DE host ncRNAs compared to macrophages from uninfected mice, and a relatively low number of *Fasciola*-derived miRNAs.

While there is an increase in the gene expression associated with the number of GO biological process pathways at 18 hrs and 5 days post-infection, macrophages at day 5 had the most uniquely enriched GO terms associated with genes that were reduced in expression by >Log2FC, correlating with the increased occurrence of both parasite and host-derived miRNAs in macrophages at these time points. At day 5, many of the cellular pathways associated with downregulated gene expression were associated with immune regulation (including ‘regulation of apoptotic signalling’; ‘MAPK cascade’; ‘regulation of immune system processes’; ‘inflammatory response’; ‘cellular response to transforming growth factor beta stimulus’; ‘leucocyte homoeostasis’; ‘defence response’).

### Host and parasite ncRNAs modulate gene expression in peritoneal macrophages to suppress pro-inflammatory responses

To determine the roles of host and parasite ncRNAs in regulating the macrophage transcriptome, target prediction analysis was performed for each of the *F. hepatica* and host DE ncRNAs (Log2FC2 cut-off) (Supplementary Tables S6.1-S6.3). Identified genes were filtered for those mRNAs that were differentially expressed in the macrophage transcriptome and present in InnateDB.com. Analysis of the gene ontology of the targeted parasite genes (Figure 7d-I; Supplementary Tables S7.1–7.3) revealed a similar number of gene targets in each pathway across all three timepoints. In contrast, for the host ncRNAs, the number of genes associated with any given cellular pathway was comparatively increased at day 5. Of note, a number of parasite and host ncRNAs were predicted to have common gene targets: Traf6, Nlrc3, Yy1, Nod1, Igf1, Hif1α, Adam17, Jam3 and Gata6. For the parasite-derived miRNAs, several of these genes (Nlrc3, Igf1, Hif1α, Gata6,) along with Pp1ca, Nod1, P2rx7, Vegfa, Nlrp6 and Plec were predicted to be targeted in macrophages at all three time-points. These genes are associated with pathways that control inflammation, such as ‘cell surface receptor signalling’, ‘MAPK cascade’, ‘canonical NF-kappaB signal transduction’, ‘cytokine production’, and ‘inflammatory response’, indicating that the fluke miRNAs specifically regulate host gene expression to dampen the innate immune response.

The data also suggests that host macrophages can detect the presence of the parasite at 6 hrs post infection as the GO terms assigned to the genes targeted by the host ncRNAs upregulated at this timepoint are associated with pathways such as ‘response to stress’; ‘response to bacterial lipopolysaccharide’; ‘response to lipid’; and ‘response to organic substance’. At the same time, downregulated genes are also predominantly associated with the inflammatory immune response (IL-6, IL-1, TNF production; ‘regulation of cytokine production’ and ‘innate immune response’), suggesting that classic pro-inflammatory responses is being regulated by host ncRNA. As the *F. hepatica* infection progresses, this suppression of innate immune pathways remains, although by day 5 post-infection, there is a notable upregulation of genes associated with a T cell response (‘T cell migration’; ‘T cell chemotaxis’; ‘activated T cell proliferation’) which coincides with the emergence of host T cell responses to the parasite as it enters the liver parenchyma [41,42]. There is also an increase in the number of cell death-related pathways activated, possibly resulting from the response of the peritoneal macrophages to the tissue damage caused as the parasite penetrates the liver capsule.

**Figure 8.**
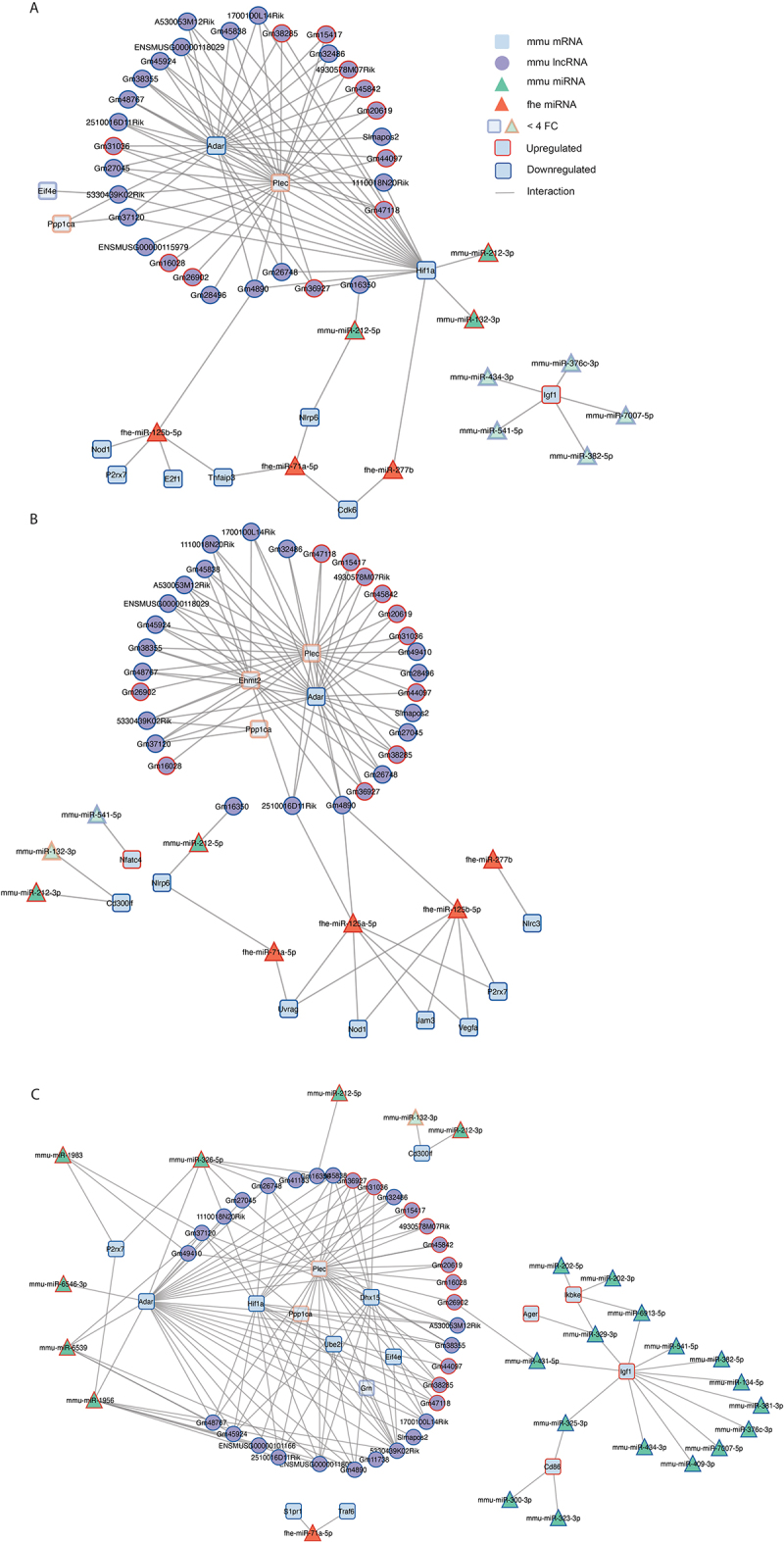
The *fasciola hepatica* vs mouse non-coding RNA interactome in peritoneal macrophages over the course of infection. This interactome represents the *F. hepatica* miRNAs (orange triangle), and differentially expressed murine miRNAs (green triangles) and murine lncRNAs (purple circle). A ± 2Log2FC cut off was applied to murine miRNAs and lncRnas. Target prediction analysis for the selected fhe-miRnas, mmu-miRNAs and lncRNAs was conducted and predicted targets were filtered for those shown to be involved in innate immunity (InnateDB.com). Blue and red borders surrounding nodes indicate downregulated or upregulated expression respectively in cells from infected mice vs uninfected animals. Transparent nodes indicate those with <±4fold differential expression. (A) 6 hrs post-infection; (B) 18 hrs post-infection; (C) 5 days post-infection.

To better understand how ncRNAs drive the regulation of these biological processes and to gain a view of the molecular networks underpinning the activation of macrophages during *F. hepatica* infection, the parasitic and host ncRNA-mRNA innate immune interactions for each timepoint were mapped in Cytoscape (Figure 8). Reflective of the RNA-sequencing data analysis, there was an increasing number of interactions as infection developed, with the largest network observed at day 5 post-infection. What was immediately clear from these interactomes is that the host ncRNA largely operates independently of the parasite ncRNA, with both targeting a separate set of genes over the course of infection.

Remarkably, the entire assembly of host lncRNAs predominantly target three mRNAs over the first 5 days of infection: Plec (upregulated), Adar1 and Hif1α (both downregulated). Hif1α is also targeted by the parasite-derived miRNAs fhe-miR-277b at 6 hrs post-infection. In addition, Nlrp6 is targeted by both mouse (miR-212-5p) and *Fasciola* (miR-71a) miRNAs at both 6 and 18 hrs post-infection. While the expression of P2×r7is decreased by *Fasciola* miRNAs at the early time points, regulation of this gene is continued by mouse derived miRNAs at day 5 post-infection. The remaining host miRNAs principally regulate the expression of Igf1 (increased in expression), which was most evident at 5 days post-infection and notably these miRNAs were primarily from the miRNA cluster on chromosome 12. The genes specifically downregulated over the course of infection by *Fasciola* miRNAs included Nod1, E2f1, Tnfaip3 and Cdk6 at 6 hrs, and Jam3, Vegfa, Nod1, Uvrag and Nlrc3 at 18 hrs. Only one *F. hepatica* miRNA was observed on day 5, fhe-miR-71, which is predicted to target Traf6 and S1pr1.

## Discussion

This thorough examination of the ncRNA:mRNA interactions within peritoneal macrophages has provided the first characterization of the molecular interplay between the host and parasite during acute infection with *F. hepatica*. Our studies suggest that both host and parasite cooperate to regulate the expression of pro-inflammatory genes, which consequently reduces the activation of a cytotoxic anti-microbial innate immune response. As a result, the parasite is tolerated by the host and can migrate from the intestine to the liver unchallenged, while repair mechanisms are quickly generated to minimize damage caused by the parasite’s movement through tissue.

The observation that host ncRNA expression in macrophages was relatively unchanged between infected and uninfected mice at 6 hrs and 18 hrs supports a scenario in which immediately after infection, it is the parasite, rather than the host, that is predominantly suppressing the activation of innate immune cells. As a result, the induction of the host’s classic innate protective anti-microbial inflammatory response is prevented/suppressed, allowing the NEJs to migrate to the liver unchallenged. This possibility was supported by our observation that none of the biological processes of macrophages during the first 18 hrs of infection were explicitly linked to immune function, instead reflecting changes in cell process and metabolism. In addition, parasite-derived ncRNAs were most abundant at these time points, coinciding with the migration of parasites through the peritoneal cavity.

Importantly, the miRNAs detected within the host macrophages in this study have all been found within the secreted EVs of *F. hepatica*, suggesting an active selection of specific miRNAs with the capacity to regulate host gene expression [33,43–45]. Furthermore, the most abundant of the parasite-derived miRNAs in macrophages, miR-125b, is conserved across mammalian species, with fhe-miR-125b being closely related to the human miR-125b, suggesting similar functionality. Indeed, it has been shown that miR-125b from *F. hepatica* and *S. mansoni* is loaded onto mammalian Ago2 within macrophages, supporting a capacity to utilize the mammalian miRNA machinery to regulate the expression of host genes [29,33]. Corroborating this biological activity, we have predicted that both fhe-miR-125b and human miR-125b target Traf-6, a transcription factor known to regulate pro-inflammatory responses, an effect which was functionally demonstrated in murine macrophages transfected with synthetic fhe-miR-125b [33].

In contrast to the early stages of infection, at day 5 a much larger difference in host miRNA was evident, suggesting an enhanced host response compared to the earlier time points. It is most likely that the penetration of the liver by the parasites at this timepoint releases signals of tissue damage that activate the peritoneal macrophages to induce reparative mechanisms essential to the health and survival of both the host and parasite. Remarkably, most of the downregulated host miRNAs at this time point were clustered on a single chromosome (12), suggesting they share a common activating signal and co-ordinate the regulation of genes that are functionally related. Chromosome 12 contains two large known miRNA clusters containing a total of 53 miRNAs [46,47]. The chr12 miRNA cluster (Chr14 in *H. sapiens*) is conserved across eutherian mammals and is embedded within the imprinted Dlk1-Dio3 domain restricted to its maternal allele and usually shows a tissue-specific expression [48]. This cluster is highly expressed in the adult brain; however, little is known about the mechanisms underlying this spatiotemporal expression [48]. To date, chr12 miRNA cluster has not been associated with disease pathways or parasitic infection; however, several of the miRNAs have been shown to regulate/activate macrophage inflammatory responses. For example, the expression of miR-369-3p inhibits the pro-inflammatory response in innate immune cells induced by bacterial endotoxin, illustrated by a significant reduction in the production of pro-inflammatory cytokines, the expression and production of inducible nitric oxide synthetase and the activation of the NLRP3 inflammasome [49]. In contrast, transfection of rodent primary macrophages with miR-379-5p induced the polarization of an M1 phenotype of macrophage, characterized by an increased expression of CD86 [50]. Similarly, the expression of miR-382-5p is significantly upregulated in human and murine LPS/IFN polarized M1 macrophages [51]. In addition, the overexpression of either miR-379-5p or miR-382-5p prevented the polarization of macrophages towards an M2(IL-4) phenotype [50,51]. The collective reduced expression of these miRNAs in the peritoneal macrophages of *F. hepatica* infected mice at day 5 indicates the presence of a mixed immune response, with the absence of miR-369-3p, expected to support the development of a pro-inflammatory response, and the absence of miR-379-5p and miR-382-5p allowing polarization towards an M2 phenotype. An increased expression of genes associated with tissue damage, tissue necrosis and immune infiltration (including macrophages) has been reported in the liver at this stage of infection with *F. hepatica* [52]. A change in the expression of this combination of host miRNAs may, therefore, reflect the combined outcome of an activation of the innate immune response to damage signals (typically pro-inflammatory M1) and the need to keep this response in check to prevent excessive cytotoxicity and ensure efficient tissue repair (typically mediated by M2 macrophages). Accordingly, this miRNA cluster may be specifically targeted by the host at this time as it has the capacity to fine-tune the immune response to damage (and infection) signals to achieve the necessary repair without significant toxicity.

Building an interactome of both host and parasite miRNAs and their respective gene targets in macrophages over the course of infection with *F. hepatica*, provided a unique insight into the collective influence of both host and parasite miRNAs on the gene regulation and thus functional activity of these cells. The three genes predicted to be reduced in expression by both parasite and host miRNAs (Hif1α, Nlrp6, P2rx7) are all associated with the activation of a pro-inflammatory macrophage phenotype. More specifically, Hif1α regulates macrophage glycolysis and is critically involved in the functional differentiation of anti-microbial, M1-like macrophages, to promote the production of pro-inflammatory cytokines and the activation of nitric oxide production [53–56]. Mediating a similar outcome, Nlrp6 (the most downregulated innate gene at 6hrs post-infection; −30.2 fold) acts as a cytosolic sensor in macrophages and mediates the secretion of pro-inflammatory cytokines IL-18, IL-1β and TNF in response to ligand stimulation [57,58]. Finally, activation of P2rx7 (the most significantly downregulated innate gene at day 5; −11-fold) induces the assembly of the NLRP3 inflammasome, resulting in the activation and secretion of the pro-inflammatory cytokines IL-18 and IL-1β [59,60]. Thus, the reduction in expression of these genes by both host and parasite miRNAs would suggest a coordinated inhibition of pro-inflammatory responses in macrophages during infection with *F. hepatica*. From the parasite’s perspective, this collective control of macrophages would reduce the immune-mediated killing of susceptible NEJs as they migrate from the intestine to the liver. Supporting this possibility, it has previously been shown that blocking the P2rx7 pathway in macrophages reduced their ability of macrophages to kill *Trichinella spiralis* larvae [61].

While some anti-microbial mediators secreted by M1-like macrophages are cytotoxic to parasites, others are ineffective against large multicellular helminths. Therefore, it is necessary for the host to minimize/regulate pro-inflammatory response to prevent bystander damage to self-tissue, tolerate the presence of the parasite, and switch to a predominant tissue repair response [62]. With this in mind, it is highly relevant that P2rx7 signalling is also reportedly reduced in macrophages of hosts infected with *S. mansoni* [63] and *Trichuris suis* [64], perhaps indicating a common mechanism of immune modulation during helminth infection. It has also been reported that mice deficient for Nlrp3 and Nlrp6 display a reduced pro-inflammatory immune response and an augmented type 2 immunity in response *T. muris* [65] or *Nippostrongylus brasiliensis* [66].

The impact on the polarization of macrophage phenotypes is evident in the function of genes that are specifically targeted by *F. hepatica* miRNAs. Of these, Nod1 is a cytoplasmic receptor that responds to signals from invading bacteria and cellular stress to activate antimicrobial mechanisms and pro-inflammatory responses in macrophages [67–69]. Similarly, the E2F transcription factor 1 is linked to the regulation of M1/M2 polarization since mice deficient in E2F1 show an enhanced abundance of M2 macrophages [70]. The suppression of expression of these genes by the parasite derived miRNAs would clearly augment the effect of the reduced expression of Hif1α, Nlrp6 and P2rx7 to suppress the development of pro-inflammatory macrophages and promote polarization towards a reparative M2-like phenotype.

Such an immune outcome is further corroborated by the extensive regulation of Igf1 by twelve mouse miRNAs in peritoneal macrophages over the duration of *F. hepatica* infection. Indeed, this gene showed the greatest fold increase in expression post-infection compared to all other innate immunity-related genes. The Igf1 hormone is secreted by IL-4 differentiated M2-like macrophages and contributes to the establishment of an anti-inflammatory environment [71]. Furthermore, an increased abundance of Igf1 has been shown to reduce bacterial translocation from the intestine [72] by promoting the proliferation and expansion of intestinal epithelial cells to restore the mucosal barrier.

The immunological effect of the widespread targeting of Adar1 and Plec by all identified host lncRNAs is less clear-cut. Although Adar1, an A-to-I RNA editing enzyme [73], has been associated with the regulation of inflammatory responses, it has been reported to both induce and inhibit the polarization of macrophages towards an M1 phenotype. The *in vivo* studies that demonstrated the induction of an M2 phenotype and reduced pro-inflammatory responses by Adar1 utilized murine models of inflammation (sepsis, allograft transplantation) in which Adar1 was overexpressed in mice following a parenteral injection of Adenovirus vector carrying the Adar1 gene [74,75]. In contrast, the specific deletion of Adar1 from macrophages was found to prevent the polarization of macrophages towards a pro-inflammatory immune response in a murine model of Abdominal Aortic Aneurysm [76]. While this discrepancy between functional outcomes is likely related to the difference in technical approaches to alter the expression of Adar1 (whole animal versus cell-specific), the reported association between Adar1 and enhanced M1 activation in macrophages aligns with the changes to other gene targets observed in the peritoneal macrophages of mice infected with *F. hepatica*. We suggest, therefore, that the increased expression of a multitude of host lncRNAs reduces the expression of Adar1 in mouse peritoneal macrophages to prevent the overactivation of the pro-inflammatory immune response to minimize tissue damage and dysfunction.

Another discrepancy exists in the published studies of the association between plectin, a cytoskeletal protein, and macrophage activation. In macrophage cell lines (J774 and RAW), the abundance of plectin was significantly increased in response to stimulation with bacterial endotoxin which impaired the production of pro-inflammatory IL-6 in response to stimulation with inflammatory ligands [77]. However, the treatment of primary murine macrophages with endotoxin did not cause an increase in plectin [78]. While the role of plectin in the polarization of macrophages thus remains unclear, the increase in its expression mediated by the host lncRNAs during *Fasciola* infection could be related to its role in structural cellular processes involving actin filament dynamics [79], such as phagocytosis and motility. This functional association is supported by the report that the abundance of plectin is specifically enhanced in dendritic cells stimulated by *S. mansoni* eggs and is linked to the profound cytoskeletal rearrangements that occur during the maturation of dendritic cells [80]. Furthermore, in mice with targeted mesenchymal ablation of plectin, there was a significant reduction in the migration of macrophages towards a skin wound, supporting the important role of plectin in the wound healing process [36]. Therefore, host lncRNAs induction of plectin may play a part in differentiating macrophages into a reparative functional phenotype during the host immune response to *F. hepatica.*

## Concluding remarks

This study has provided added insight into the possible mechanisms by which helminth parasites alter the host innate immune response to change the functional activity of macrophages, enhancing their survival. The additional finding that this regulation of pro-inflammatory pathways is supported by host ncRNA confirms the long-held hypothesis that rather than attempt to expel the parasite, the host tolerates their presence and focuses the biological activity of immune cells on tissue repair.

The identification of specific gene targets for the parasite miRNAs highlights potential pathways that, if induced during infection, may mediate killing and/or expulsion. With the emergence of target site blockers, this could be tested in vivo, which would ascertain whether interfering with the functions of parasite miRNAs and/or manipulating the expression of their gene targets represents a new strategy to achieve therapeutic control of these pathogens, and regulation of mammalian inflammatory responses. At a more fundamental level, a deeper understanding of the selection and packaging of parasite miRNAs into EVs will be critical to fully elucidate the mechanisms of Ago-loading, functionality and cross-communication with host cells that parasites have evolved to adapt to life within their mammalian hosts.

## Supplementary Material

Sais et al_Supplementary Table 6.xlsx

Sais et al_Supplementary Table 5.xlsx

Sais et al_Supplementary Table 3.xlsx

Sais et al_Supplementary Table 2xlsx.xlsx

Sais et al_Supplementary Table 7.xlsx

Sais et al_Supplementary Table 1.xlsx

Sais et al_Supplementary Table 4.xlsx

## Data Availability

The total RNA-seq and miR-seq data that underpin the analysis presented in this study is freely available and deposited in National Center for Biotechnology Information (NCBI) Gene Expression Omnibus; accession number GSE263895. All data generated or analysed during this study are included in this published article [and its supplementary information files].
